# Anomalies de l’électro-encéphalogramme en neurologie pédiatrique: à propos de 500 enregistrements à l’Hôpital Gynéco-Obstétrique et Pédiatrique de Yaoundé (Cameroun)

**DOI:** 10.11604/pamj.2013.15.63.2765

**Published:** 2013-06-21

**Authors:** Séraphin Nguefack, Dominique Enyama, Andreas Chiabi, Victor Sini, Evelyn Mah, Jean Baptiste Bogne, Paul Cédric Mbonda, Elie Mbonda

**Affiliations:** 1 Service de pédiatrie et sous spécialités pédiatriques, Hôpital Gynéco-obstétrique et Pédiatrique de Yaoundé; 2 Département de pédiatrie, Faculté de Médecine et des sciences Biomédicales; 3 Service de neurologie, Hôpital Central de Yaoundé, Cameroun

**Keywords:** Enfants, épilepsies, électro-encéphalogramme, Cameroun, children, epilepsies, EEG, Cameroon

## Abstract

**Introduction:**

Cette étude dont le but était d’évaluer la contribution de l’électroencéphalogramme (EEG) en neurologie pédiatrique et de déterminer les indications pertinentes chez l’enfant de 0 à 15ans.

**Méthodes:**

Il s’agit d’une étude rétrospective et descriptive réalisée au laboratoire d’électroencéphalographie de l’Hôpital Gynéco-Obstétrique et Pédiatrique de Yaoundé du 1er novembre 2011 au 15 mars 2012.

**Résultats:**

L’âge moyen des patients était de 70.2 mois avec des extrêmes de 0 et 180 mois. Le sexe ratio était de 1.04. Cent quatre vingt treize des 500 tracés de veille étaient anormaux 41 des 114 tracés de sommeil étaient anormaux et 78 des 500 tracés réalisés présentaient un rythme de fond ralenti pour l’âge. Cent cinquante tracés présentaient des anomalies épileptiques dont 81 focales, 35 multifocales et 34 des anomalies généralisées. Sur les 137 patients dont l’EEG était compatible avec une épilepsie, le lobe temporal était le plus souvent le siège d’anomalies épileptiques avec des épilepsies temporales et des épilepsies à pointes centro-temporales, venaient ensuite le lobe frontal, les épilepsies généralisées, les épilepsies du lobe occipital et l’hypsarythmie. Chez 13 des 150 patients avec des anomalies épileptiques à l’EEG, les anomalies retrouvées ne rentraient pas dans le cadre d’un syndrome épileptique particulier. Lorsque l’épilepsie était connue, la probabilité d’avoir un tracé EEG anormal était 1,44 fois plus élevée (OR=1.44 (0.83-2.52) même si la corrélation n’était pas statistiquement significative (p=0.1). En revanche lorsque l’épilepsie était suspectée, il y avait 3.43 fois plus de risques d’avoir un tracé anormal (OR=3.43 (2.27-5.18) avec une corrélation statistiquement significative (p< ;0.05). Les convulsions fébriles, les mouvements anormaux, le retard psychomoteur, les troubles déficitaires de l’attention avec hyperinésie, la perte de connaissance et les troubles du langage n’étaient pas significativement corrélés avec un risque accru d’avoir un EEG anormal.

**Conclusion:**

L’EEG a un rôle aussi bien dans la confirmation et la caractérisation de divers syndromes épileptiques et suspicions d’épilepsie que dans la discrimination des manifestations paroxystiques non épileptiques chez l’enfant. Les renseignements cliniques sont indispensables pour une lecture optimale du tracé.

## Introduction

Depuis la découverte de l’électroencéphalogramme humain (EEG) en 1924 par Berger, il constitue un outil important dans le diagnostic de diverses affections neurologiques chez l’enfant [1, 2]. Il permet non seulement de faire le diagnostic des épilepsies et de diverses affections cérébrales, mais aussi d’en distinguer les manifestations paroxystiques non épileptiques [3–5]. L’innocuité de l’examen facilite son Utilisation, et son usage excessif. La plupart des travaux réalisés sur l’électroencéphalographie chez l’enfant ont évalué l’apport de l’EEG dans le diagnostic des syndromes épileptiques et chez des enfants présentant des signes et symptômes neurologiques spécifiques tels que les céphalées [6, 7], les troubles déficitaires de l’attention avec hyperkinésie [8], des syncopes et des mouvements anormaux [9].

Le Cameroun, avec une population de 20 millions d’habitants, ne dispose que d’une douzaine d’appareils d’électroencéphalographie concentrés dans deux villes du pays (Yaoundé et Douala). Ce faible ratio d’appareils d’électroencéphalographie par rapport au nombre d’habitants, l’inégale répartition sur le territoire national des appareils existants et le coût de l’examen rendent compte de la difficile accessibilité à l’EEG, tant géographique que financière, dans notre milieu. Ce travail, réalisé au laboratoire d’EEG de neurologie pédiatrique et d’épileptologie de l’Hôpital Gynéco-Obstétrique et Pédiatrique de Yaoundé, avait pour but d’évaluer la contribution de l’EEG en neurologie pédiatrique et de déterminer des indications pertinentes dans un contexte ù son accessibilité est limitée. Notre objectif général était de décrire les anomalies électroencéphalographiques retrouvées en neurologie pédiatrique et plus spécifiquement de déterminer la fréquence des tracés anormaux et la fréquence des tracés EEG compatibles avec une épilepsie ainsi que la corrélation entre les indications cliniques et les tracés anormaux.

## Méthodes

Il s’agissait d’une étude rétrospective et descriptive menée du 1^er^ novembre 2011 au 15 mars 2012 au laboratoire d’EEG de l’Hôpital Gynéco-Obstétrique et Pédiatrique de Yaoundé. Nos critères d’inclusion étaient tous les patients âgés de 0 à 15 ans avec une demande d’examen spécifiant l’âge, le sexe et les renseignements cliniques.

Tous ces patients ont bénéficié d’un enregistrement électroencéphalographique réalisé par une unité d’acquisition EEG de 32 canaux de Micromed-France. Le montage utilisé était conforme au système international 10/20 avec mise en place de 10 ou de 21 électrodes selon l’âge du patient et son périmètre crânien. La stimulation lumineuse intermittente (SLI) a été réalisée chez tous les patients tandis que l’hyperpnée n’a été réalisée que chez les enfants de plus de 3 ans, chaque fois que cela était possible (coopération de l’enfant et absence de contre indication). Chez les patients suspects d’épilepsie absence, une hyperpnée prolongée (2 fois 3 minutes) pouvait être pratiquée si l’épreuve classique n’induisait pas de crise. Tous les enregistrements ont été réalisés par un technicien et un compte rendu selon un modèle standardisé comportant la date de naissance, la date d’examen, le nom du médecin prescripteur, les renseignements cliniques, la description du rythme de fond, la description d’anomalies paroxystiques spontanées ou provoquées lors des épreuves d’activation et une conclusion. Toutes les données ont été collectées à partir des comptes rendus d’examens et colligées de manière anonyme à l’aide d’une fiche technique pré établie comportant les données générales relatives au patient, les renseignements cliniques et les résultats de l’enregistrement. Avant le début du travail, nous avons obtenu l’autorisation du comité éthique institutionnel de l’Hôpital Gynéco-Obstétrique et Pédiatrique de Yaoundé.

Les données recueillies à l’aide du questionnaire ont été collectées dans une base de données Access^®^ et traitées à l’aide du logiciel d’analyse statistique Epi-info^TM^ version 3.5. Les logiciels de saisie Word et Excel version 2007 de Microsoft^®^ ;ont permis la réalisation des tableaux et figures, ainsi que le traitement de texte. La différence entre les associations a été évaluée à l’aide des Odds Ratio (OR) avec un intervalle de confiance à 95%. La valeur de p < ;0.05 a été considérée comme statistiquement significative.

## Résultats

**Figure 1:**
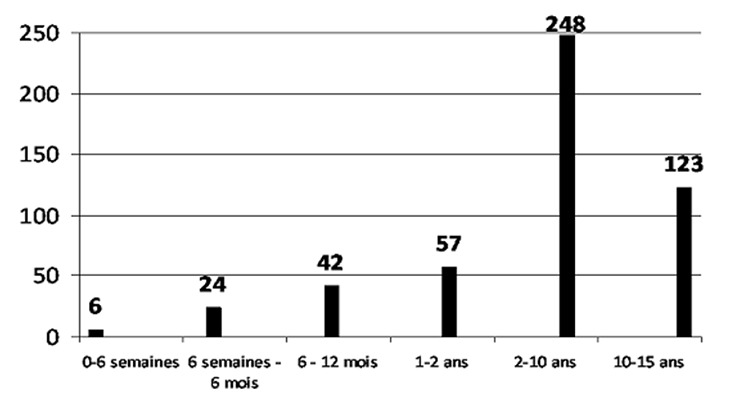
Répartition par tranche d’âge

Durant la période d’étude, 556 tracés ont été réalisés au laboratoire d’EEG de l’HGOPY, parmi ces tracés 500 répondaient aux critères d’inclusion. L’âge moyen des patients était de 70.2 mois (±;55.6 mois) avec des extrêmes de 0 et 180 mois. Le sexe ratio était de 1.05. La Figure 1 montre la répartition des patients en fonction des tranches d’âge.

**Figure 2:**
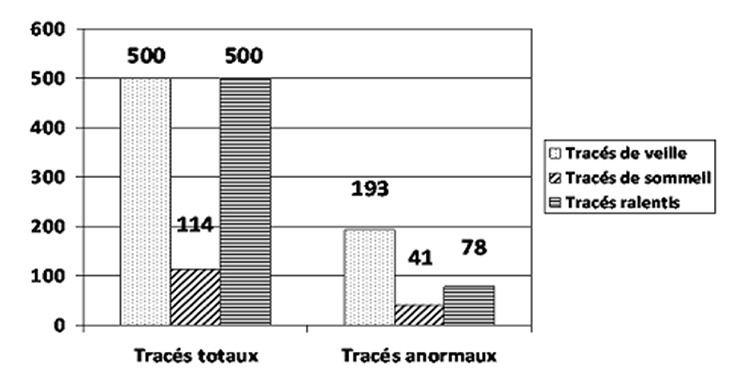
Fréquence des tracés anormaux

**Figure 3:**
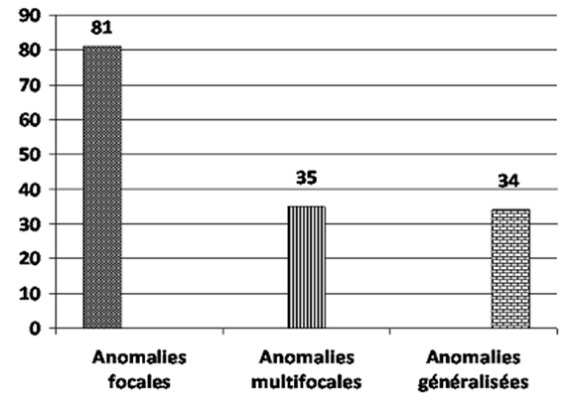
Répartition des anomalies épileptiques

**Figure 4:**
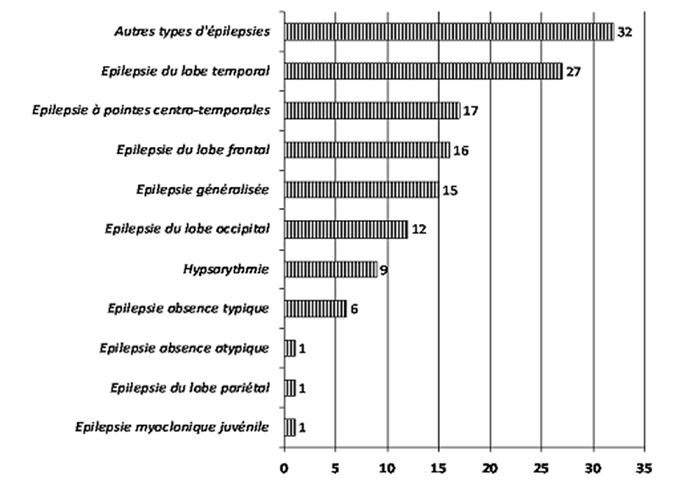
Fréquence des diagnostics EEG des épilepsies

La Figure 2 montre la répartition des tracés EEG anormaux en fonction du type de tracé (veille, sommeil), ainsi que la fréquence des tracés lents. Ainsi, 193 des 500 tracés de veille étaient anormaux, 41 des 114 tracés de sommeil étaient anormaux et 78 des 500 tracés réalisés présentaient un rythme de fond lent pour l’âge. La Figure 3 montre la répartition des anomalies épileptiques rencontrées. Sur les 500 enregistrements étudiés, 150 présentaient des anomalies épileptiques. Sur les 500 tracés analysés, 137 patients avaient un diagnostic EEG d’épilepsie. La répartition des épilepsies diagnostiquées par l’EEG est détaillée dans la Figure 4.

**Figure 5:**
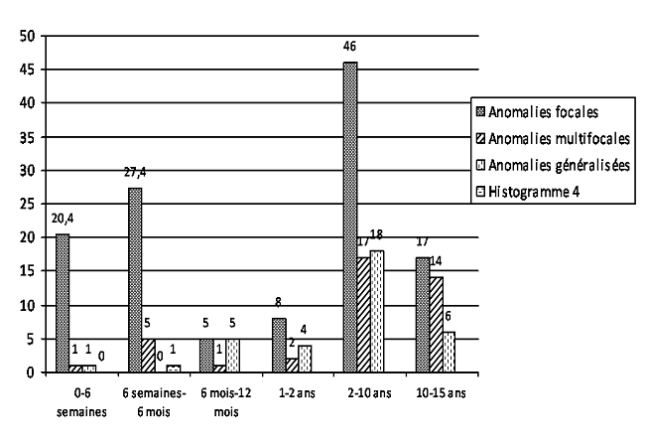
Répartition des anomalies épileptiques retrouvées à l’EEG en fonction des tranches d’âge

Concernant les indications cliniques des demandes d’EEG on a trouvé que: 56 patients sur 500 (11.2%) avaient un EEG demandé dans le cadre d’une épilepsie connue dont 22 (39.4%) présentaient des anomalies épileptiques enregistrées; 134 patients sur 500 (26.8%) avaient un EEG demandé dans le cadre d’une épilepsie suspectée dont 73 (54.5%) présentaient des anomalies EEG enregistrées. Le Table 1 récapitule la fréquence des anomalies retrouvées à l’EEG chez les patients avec une suspicion d’épilepsie d’une part et chez les patients avec une épilepsie confirmée d’autre part. Le Table 2 montre les corrélations entre les renseignements cliniques portés sur la demande d’examen et la fréquence des tracés EEG anormaux. La Figure 5 montre la répartition des différentes anomalies épileptiques retrouvées à l’EEG en fonction des tranches d’âge.

**Tableau 1:** Fréquence des anomalies EEG retrouvées en cas d’épilepsie connue et suspectée

Epilepsie connue Vs suspectée	Anomalies focales	Anomalies multifocales	Anomalies généralisées	Tracés lents
Epilepsie connue	11/22 (50%)	8/22 (36.4%)	3/22 (13.6%)	8/56 (14.3%)
Epilepsie suspectée	33/73 (45.2%)	14/73 (19.2%)	26/73 (35.6%)	19/134 (14.2%)

**Tableau 2:** Corrélations entre indications cliniques et résultat d’EEG

Indication clinique d’EEG	Tracé anormal*	Odd ratio	p-value
Epilepsie connue	27/56 (48.2%)	1.44 [0.83-2.52]	0.1
Epilepsie suspectée	83/134 (61.9%)	3.43 [2.27-5.18]	0.00
Convulsions fébriles	2/28 (7.1%)	0.1 [0.002-0.44]	0.00
Mouvements anormaux	6/25 (24%)	0.45 [0.17-1.15]	0.04
Troubles de l’attention avec hyperactivité	1/3 (33.3%)	0.74 [0.06-8.24]	0.43
Retard psychomoteur	13/40 (32.5%)	0.69 [0.35-1.38]	0.15
Perte de connaissance	3/34 (8.8%)	0.13 [0.004-0.43]	0.00
Troubles du langage	2/5 (40.0%)	0.99 [0.16-5.98]	0.49
Troubles de conscience	1/1 (100.0%)	Indéfini	0.2
Céphalées	0/4 (0.0%)	0	0.06
Hystérie	0/11 (0.0%)	0	0.0001
Difficultés scolaires	0/3 (0.0%)	0	0.01
Troubles du sommeil	0/2 (0.0%)	0	0.17
Troubles de l’apprentissage	Aucun patient	-	-

* Tracé anormal = tracé avec des anomalies et/ou tracé lent

## Discussion

Cette étude suggère que deux indications cliniques semblent plus souvent associées à un EEG anormal: la suspicion d’épilepsie qui est associée à 61.9% de tracés anormaux avec un Odd ratio à 3.43 (p < ;0.05) et l’épilepsie connue, indication associée à 48.2% de tracés anormaux avec un Odd ratio à 1.44 (0.83-1.25) (p=0.1). Ces deux indications cliniques nous semblent être les plus pertinentes pour motiver une demande d’électroencéphalogramme dans notre contexte.

La répartition par tranches d’âge montre une proportion importante d’enfants dans les tranches d’âge de 2 à 10 ans et de 10 à 15 ans avec respectivement 248 et 123 patients sur 500. Les nouveau-nés et les nourrissons jusqu’à 6 semaines représentent une proportion minoritaire des patients avec 6 patients sur 500.

Deux cent un tracés sur 500 soit 40.2% des tracés étaient anormaux. Cent cinquante d’entre eux présentaient des anomalies de nature épileptique soit 30% des patients. Kürsad en 2003 en Turquie avait retrouvé 36.2% de tracés anormaux dans une série de 534 patients, ce qui est sensiblement identique à nos trouvailles [11]. Tous les tracés de notre série (n=500) étaient de veille et 114 étaient de sommeil spontané. Ces résultats sont similaires à ceux retrouvés par Kürsad à Ankara en 2003 qui sur 534 tracés retrouvait 415 tracés de veille, 100 tracés de sommeil spontané et 19 tracés de sommeil après privation de sommeil [11].

L’étude des anomalies épileptiques retrouvées a révélé: des anomalies focales, multifocales et généralisées respectivement chez 54% (81/150), 23.3% (35/150) et 22.7% (34/150) des patients. Sur les 137 patients dont l’EEG était compatible avec une épilepsie, le lobe temporal était le plus souvent le siège d’anomalies épileptiques avec des épilepsies temporales (27/137) et des épilepsies à pointes centro-temporales (17/137), venaient ensuite le lobe frontal (16/137), les épilepsies généralisées (15/137), les épilepsies du lobe occipital (12/137) et l’hypsarythmie (9/137). Chez 13 des 150 patients avec des anomalies épileptiques à l’EEG, les anomalies retrouvées ne rentraient pas dans le cadre d’un syndrome épileptique particulier.

Jan Mohamed à Jeddah [1] en 2002 avait retrouvé sur 138 patients des épilepsies du lobe temporal (10/138), des épilepsies à pointes centro-temporales (8/ 138). Ceci représente proportionnellement moins d’atteintes du lobe temporal que celles observées dans notre série mais ces dernières restaient l’atteinte majoritaire avec 18 patients sur 138.

Les épilepsies du lobe frontal (17/138), les épilepsies généralisées (15/138) et l’hypsarythmie (9/138) étaient également représentées dans l’étude de Jan Mohamed et dans la notre [1]. Les épilepsies du lobe occipital (5/138), étaient moins fréquentes dans l’étude de Jan Mohamed que dans la notre [1]. Ceci montre que la répartition des épilepsies de l’enfant reste globalement comparable d’une étude à l’autre.

Les EEG des 56 patients avec une épilepsie connue (11.2% de notre population d’étude) ont révélé des anomalies épileptiques chez 22 d’entre eux. Ces anomalies étaient réparties en anomalies focales, multifocales et généralisées avec respectivement 50%, 36.4% et 13.6%. En outre, 14.3% des patients avec épilepsie connue avaient un tracé EEG ralenti pour l’âge. Cent trente quatre EEG demandés dans le cadre d’une suspicion d’épilepsie (26,8% de notre population d’étude) dont 73 présentaient des anomalies épileptiques soit 54.5%. L’analyse détaillée du type d’anomalie retrouvée a montré: 45.2% d’anomalies focales, 19.2% d’anomalies multifocales et 35.6% d’anomalies généralisées tandis que le tracé EEG était ralenti chez 14.2% des patients.

Cette tendance plus élevée d’anomalies épileptiques à l’EEG était déjà rapportée par Kürsad en 2003 qui retrouvait une prépondérance d’anomalies EEG chez les patients avec épilepsie suspectée (33.8%) comparativement aux patients avec épilepsie établie (31.2%) [11]. Israel Matoth et al. à Jérusalem en 2002 sur 547 patients a retrouvé une tendance similaire à la notre avec des anomalies épileptiques chez 43% d’enfants épileptiques connus et une fréquence plus importante en cas d’épilepsie probable (53%) [9].

En revanche Jan Mohamed à Jeddah en 2002 sur une série de 438 malades avait retrouvé des anomalies à l’EEG chez 43% des enfants épileptiques connus contre 35% d’enfants avec une suspicion de comitialité [1].

Notre étude suggère que les patients adressés pour suspicion d’épilepsie présentent une fréquence plus élevée d’anomalies à l’EEG comparativement aux patients épileptiques connus (54.5% versus 39.4%). Ces résultats pourraient s’expliquer par le fait que l’essentiel des demandes d’EEG chez les patients épileptiques connus est faite en vue de l’arrêt du traitement antiépileptique, diminuant de fait l’incidence des anomalies épileptiques chez ce groupe de patients.

Les convulsions fébriles (7,1%), les mouvements anormaux (24%), le retard psychomoteur (32,5%), les troubles avec déficit de l’attention et hyperactivité (33,3%), la perte de connaissance (8,8%) et les troubles du langage (40%) n’étaient pas significativement corrélés avec un risque accru d’avoir un tracé anormal. Les patients présentant des céphalées (n=4), un syndrome de conversion hystérique (n=11), des difficultés scolaires (n=3) et des troubles du sommeil (n=2) avaient un tracé EEG normal.

Israel Matoth et al. à Jérusalem en 2002 sur 547 patients a retrouvé 29% de tracés anormaux chez les enfants présentant des troubles avec déficit de l’attention et hyperactivité, 5% de tracés anormaux chez des enfants avec des céphalées [9]. Kürsad Aydin à Ankara en 2003 sur 534 tracés avait retrouvé 15% de tracés anormaux dans les convulsions fébriles, 6.1% chez des enfants avec troubles déficitaires de l’attention et hyperactivité, 4.6% chez dans les céphalées, 12.5% dans les troubles du sommeil [11]. Néanmoins ces résultats montrent qu’il existe une grande disparité de résultats EEG dans les indications cliniques autres que l’épilepsie suspectée et connue.

Kramer et al. en 1994 retrouvait une activité épileptiforme chez 12% et un ralentissement focal ou généralisé chez 8,6% des patients présentant des céphalées chroniques [7]. Gronseth et Greenberg [12] dans une revue de 40 articles décrivant les trouvailles EEG chez des patients avec des céphalées avaient démontré que l’EEG n’était pas un outil diagnostic efficace pour déterminer la cause des céphalées. Nos résultats suggèrent bien que l’EEG n’est pas utile dans l’exploration des céphalées de l’enfant en routine. Cette étude sur la contribution de l’électroencéphalogramme en neurologie pédiatrique au Cameroun offre une vision synoptique du problème et ouvre de nombreuses voies pour des recherches ultérieures. Des travaux pour affiner les différents types d’épilepsies diagnostiquées à l’EEG, ou encore pour préciser l’intérêt de l’EEG chez le nouveau-né et le jeune nourrisson seraient intéressants à mener.

## Conclusion

Au terme de cette étude, il ressort que l’électroencéphalogramme garde une place importante dans la pratique quotidienne en neurologie pédiatrique dans notre contexte. Il a un rôle aussi bien dans la confirmation et la caractérisation de divers syndromes épileptiques et suspicions d’épilepsie que dans la discrimination des manifestations paroxystiques non épileptiques chez l’enfant. Les renseignements cliniques sont indispensables pour une lecture optimale du tracé.
